# Coordination dynamics of multi-agent interaction in a musical ensemble

**DOI:** 10.1038/s41598-021-04463-6

**Published:** 2022-01-10

**Authors:** Shannon Proksch, Majerle Reeves, Michael Spivey, Ramesh Balasubramaniam

**Affiliations:** 1 Cognitive and Information Sciences, University of California-Merced, Merced, USA; 2 Applied Mathematics, University of California-Merced, Merced, USA

**Keywords:** Psychology, Human behaviour

## Abstract

Humans interact with other humans at a variety of timescales and in a variety of social contexts. We exhibit patterns of coordination that may differ depending on whether we are genuinely interacting as part of a coordinated group of individuals vs merely co-existing within the same physical space. Moreover, the local coordination dynamics of an interacting pair of individuals in an otherwise non-interacting group may spread, propagating change in the global coordination dynamics and interaction of an entire crowd. Dynamical systems analyses, such as Recurrence Quantification Analysis (RQA), can shed light on some of the underlying coordination dynamics of multi-agent human interaction. We used RQA to examine the coordination dynamics of a performance of “Welcome to the Imagination World”, composed for wind orchestra. This performance enacts a real-life simulation of the transition from uncoordinated, non-interacting individuals to a coordinated, interacting multi-agent group. Unlike previous studies of social interaction in musical performance which rely on different aspects of video and/or acoustic data recorded from each individual, this project analyzes group-level coordination patterns solely from the group-level acoustic data of an audio recording of the performance. Recurrence and stability measures extracted from the audio recording increased when musicians coordinated as an interacting group. Variability in these measures also increased, indicating that the interacting ensemble of musicians were able to explore a greater variety of behavior than when they performed as non-interacting individuals. As an orchestrated (non-emergent) example of coordination, we believe these analyses provide an indication of approximate expected distributions for recurrence patterns that may be measurable before and after truly emergent coordination.

## Introduction

Science has looked to art for inspiration in explaining human cognition. Music, in particular, has aided scientists exploring human engagement with the world, from emotional experience^1–3^ to social interaction^4–8^. Music provides an ideal model system of human social interaction—balancing ecological validity of the interaction and environment with experimental control^4^.

Consider the initiation of the slow clap by one, then two, then four people, before breaking into full audience applause or the first musician in a flash mob initiating a flow of musicians and audience members engaging in shared music making. The truly emergent sound of audience applause, and the script-guided pseudo-emergent sound of a musical flash mob each provide examples of acoustic behavioral patterns showcasing the transition from individual behavior to multi-agent interaction. Studying the patterns which arise from pseudo-emergent coordination aided by a musical script can shed light on some of the coordination dynamics which underlie truly emergent multi-agent human interaction.

Transitions from disorder to order are exhibited by a variety of animals ranging from locusts marching^9^ to birds flocking^10^ to humans clapping^11^. The patterns and conditions for this emergent coordination between individuals has been a subject of laboratory study for decades. Spontaneous, or emergent, patterns of entrainment are measured between individuals by analyzing video and motion capture from interacting dyads swinging pendulums^12^ or rocking in rocking chairs^13^. Recent work has evaluated motor coordination dynamics of naturalistic interactions such as interactive problem solving^14,15^, naturalistic conversation between individuals^16,17^, speed-dating partners^18^, and motor and acoustic coordination of performing musicians in duets^5,7^ and larger ensembles^6,8^. In these interactions, behavioral output of each interacting individual was measured and analyzed for meaningful correlations *between individuals*. An investigation of emergent synchrony in audience applause explicitly measured acoustic output of the group^11^. However, the motor behavioral patterns were still measured from the *local* behavior of *individual* audience members in order to evaluate correlation with the global signal of the audience. In each of these studies, it has been possible to obtain clear measurements of individual behavior to examine the emergent coordination dynamics of multi-agent interaction and social self-organization. However, if that multi-agent group has indeed self-organized into a complex system, then the interdependence between the agents’ functions should make it possible to detect that coordination from almost any time series emitted from that system, using state-space reconstruction^19,20^. Thus, if obtaining movement or acoustic measurements at the individual and local level is not feasible or practical with group behaviors in the wild, then global-level measurements should suffice. One of the simplest global measures to employ is an acoustic recording taken from a well-placed microphone. Increasingly, naturalistic recordings from e.g. Youtube are being used in human behavioral research where specific recording equipment is unknown, and likely does not contain visual or auditory signals mapped to individual-level behavior in group settings. Benefits of these sorts of recordings include heightened ecological validity and real world behavior. Already, Alviar et al 2020 and Kello et al 2017 analysed coordination between sound and movement, and multiscale structure in orchestral music, jazz, TED talks, and even animal vocalizations through a collection of videos found on youtube (and other corpora) with ostensibly varying recording setups, number and type of microphones, etc^21,22^.

This paper shows that it is possible to describe coordination patterns of multi-agent interaction by analyzing a time series extracted solely from group-level audio data. Rather than analyzing the individual-level behavior of interacting agents, we used nonlinear methods from the dynamical systems framework on *group-level acoustic data*. We analyzed global patterns of coordination in a musical performance of “Welcome to the Imagination World”^23^. This performance enacts a physical simulation of an orchestrated (non-emergent) transition from uncoordinated to coordinated interaction. We used Recurrence Quantification Analysis (which relies on state-space reconstruction) to investigate patterns of coordination from the audio signal of a performance of this work^24^. Although this analysis is being applied on a single recording, we believe recurrence measures of this orchestrated musical performance provide an indication of the possible expected distributions for recurrence patterns that may be observable before and after spontaneous emergent coordination. Moreover, analysis of the transition itself from uncoordinated to coordinated behavior may provide insight into the trade-off between the playful enjoyment of novelty and the rigor of predictive success^25^. Finally, we discuss applications to other examples of real-world multi-agent human interactions, such as multi-agent interaction at sporting events or of individuals coordinating in a protest.

## Human behavior as a complex dynamical system

### Principles of complex dynamical systems

A canonical example of a simple dynamical system is the pendulum clock. A pendulum is a mechanical device—e.g. a fixed weight on a string—which oscillates isochronously around a central point, meaning that swings in both directions take equal amounts of time. The consistent rate of oscillation made the pendulum clock an ideal time-keeper following its invention in the seventeenth century by Cristian Huygens. The pendulum clock and the metronome are examples of simple dynamical systems. The state (location) of the pendulum at any given time is determined by the trajectory of the pendulum over historical time. The oscillatory behavior of a pendulum can be explained by a system of differential equations.

What is relevant here is the behavior that emerges among two or more pendulums placed on a shared surface. Huygens observed that two pendulums hanging from a single beam will spontaneously—or emergently—synchronize their behavior, swinging simultaneously in anti-phase with one another. Multiple metronomes placed atop a platform balanced on two cylinders will also demonstrate emergent synchronized behavior^26,27^. A metronome is a special type of pendulum, which clicks at isochronous intervals to aid time-keeping for musicians. Metronomes feature a fixed weight at the base of a rod, in addition to a moveable weight which slides along the top of the rod to adjust the speed of the metronome oscillations, and thus the speed (tempo) of the audible metronome clicks. If set at the same tempo, the oscillating pendulum and audible clicks of multiple metronomes will begin to synchronize both in-phase and anti-phase with one another^27^. What begins as multiple individual metronome clicks will transition to clicks occurring simultaneously, as globally isochronous acoustic events. For both the pendulum clocks and the clicking metronomes, the local behavior of each individual pendulum is coupled to the behavior of the surrounding pendulums due to their behavior within a shared context — in this case a physical connection via a single beam or a single platform. The local behavior of each individual metronome or pendulum (each oscillating at approximately the same frequency but different phase) eventually self-organizes into *emergent* global patterns of synchronized behavior.

We observe similar patterns of behavioral synchronization in multi-agent human interaction. The shared context for multi-agent human behavior need not be a physical connection like the metronomes’ shared platform. Rather, the shared context mediating emergent global patterns of human behavior is the *interaction* itself. When local behavior of individual human agents becomes coupled to the behavior of surrounding agents—via the shared context of interaction—the agents self-organize to exhibit emergent global patterns of coordinated behavior. We can detect this emergent coordination of multi-agent human groups from their acoustic behavior over time, just as we can detect the emergent coordination of metronomes from their acoustic output over time.

### Principles of recurrence quantification analysis

Multi-agent human behavior, such as in crowds or musical ensembles, can be considered as complex dynamical systems, where complex global patterns of behavior emerge as a result of the self-organization of individual agents over time acting according to simple local rules. The behavior of such a dynamical system can be visualized in recurrence plots. These recurrence plots display the system’s trajectory through a phase-space, depicting when that trajectory revisits locations within that phase-space at each moment in time. Recurrence quantification analysis is used to describe the complexity of a system over time by analyzing small-scale structures in the recurrence plot.

There are a few key concepts underlying the generation of recurrence plots from time series data. In a recurrence plot, the time series data will sit on a plot with axes of time by time. A point (*i, j*) is plotted if the value at time *i* and time *j* are sufficiently similar — that is, *recurrent* — within a specified neighborhood size of the N-dimensional state-space^28^. The state-space of a dynamical system is the vector of possible combinations of states in some number of observable and unobservable dimensions. In order to determine which points are recurrent, it is necessary to reconstruct the higher dimensional phase space of the system. *State-space reconstruction* is done by embedding the original time series against a time lagged copy of itself^29^. Each time lagged copy is an additional *embedding dimension* within the state-space^30^. Takens’ theorem (1981) shows that the coupling of activity between dimensions preserves the information dynamics of the system as a whole in any single dimension. Put another way, because the subcomponents of a complex dynamical system are intrinsically interdependent, a measurement taken from any one observable subcomponent encodes information from every other (potentially unobservable) subcomponent in the system. Thus, reconstruction of the N-dimensional state-space from a single measured time series allows us to infer the topological dynamics of a multivariate system because the influence of higher dimensional dynamics is encoded in the measured dimension^31^.

The logic of state-space reconstruction and embedding within higher dimensions is important for evaluating the dynamics of a natural complex system. The dynamics of natural systems such as crowd behavior, sounds within a piece of music, or even weather patterns, contain N possible state variables as well as N possible combinations of nonlinear and bidirectional interactions. State-space reconstruction allows us to infer these unmeasured or unobservable higher order dynamical variables from a single measured variable, in order to evaluate the characteristic dynamics of a system’s behavior over time. For a weather system, we might measure the flow of high- and low-pressure systems to evaluate the transitions from stable, “good” weather to instability that precedes a storm. In a crowd or a musical performance, we might measure movement or acoustic signal to investigate the higher order dynamics of transitions between periods of instability and incoherence to periods of stability and coordination. Here, we take a simple global measure of acoustic signal recorded from a musical performance. This performance demonstrates an orchestrated (non-emergent) transition from uncoordinated to coordinated interaction (described in more detail below). We use RQA to investigate the patterns of coordination in the audio signal as represented in small-scale structures within recurrence plots. We focus on five key measures: recurrence rate, determinism, entropy, average diagonal length, and laminarity.

## Music and multi-agent human intearction: a model from acoustic data

### Musical ensembles as models of human social interaction

Music provides an ideal model system of human social interaction by providing a balance between ecological validity of the social interaction and experimental control (p111)^4^.

Analysis of social interactions in musical performance are aided by a “script-like description of the interaction” via the musical score that can be manipulated or referenced by researchers examining the behavioral dynamics of the interaction (p112)^4^. Applying methods of Granger-causality to motion capture data of individual musicians within a performing string quartet can be used to investigate how predictive the history of behavior of one musician is for the future behavior of another. The bodily sway dynamics of these interacting musicians carries Granger-causal information about leader and follower behavior of each musician^6^, and Granger-coupling of bodily sway also carries information about the joint emotional expression and perceived emotional intensity of a musical performance^8^. Even without reference to a strict musical score, studies of musical interaction have provided insight into how we anticipate and adapt to the behavior of other individuals. Nonlinear analysis techniques have revealed spontaneous self-organizing patterns of coordination across a variety of timescales during joint musical improvisation without a strict score. In a series of experiments analyzing interactions between improvising musicians, Walton et al. describe how behavior produced and received from both the kinesthetic and sonic domain serves to influence and constrain mutual improvisers from the lens of complex dynamical systems^5,7^ . Using cross-wavelet spectral analysis and Recurrence Quantification Analysis, Walton et al. describe how mutual behavioral constraints enable an improvising ensemble to produce more complex patterns than any individual would otherwise^5,7^. This mutual interaction establishes a single synergetic system at the level of the improvising group, rather than a set of individuals behaving as single agents.

Rather than analyzing the individual-level behavior of interacting musicians, we apply dynamical systems analysis— specifically RQA— on *group-level acoustic data* to analyze global patterns of coordination in a musical performance. We chose to analyze this performance because it enacts a phenomenological simulation (described below) of the transition from the uncoordinated behavior of individuals to coordinated group behavior that mimics naturalistic multi-agent human interaction.

The composition “Welcome to the Imagination World” composed by Daisuke Shimizu for wind orchestra serves as the model system for multi-agent human interaction. Specifically, the interaction of interest is the shift in dynamics from an uncoordinated, incidental collection of musicians, to a coordinated, interacting ensemble. This transition from uncoordinated, to coordinated interaction is evident in the phenomenological experience of attending (or indeed, performing) this piece of music. The audience will note that, at first, the musicians on stage have no conductor. They sound and look like they are each playing their individual warm up routine. This is because, in fact, the musicians’ score tells them to play at random. The composer wanted the sound to be aleatoric, or to occur by chance without being strictly composed. This uncoordinated soundscape continues until a melodic pattern starts to emerge from a few of the musicians, still in the absence of a conductor, and still not appearing to be coordinating with the other performers. Next, the “conductor walks on stage [as] the horn, tenor and bass instruments unify into a majestic introduction”, according to program notes from the composer (windrep.org). This marks the transition from the uncoordinated actions of individual musicians to the coordinated ensemble musicianship the audience expects. The remaining musical score is composed to dictate the acoustic interaction of the musicians on stage. Thus, the rest of the performance demonstrates the coordinated interactions of an interdependent complex system: the multi-agent musical ensemble.

## Results

**Figure 1:**
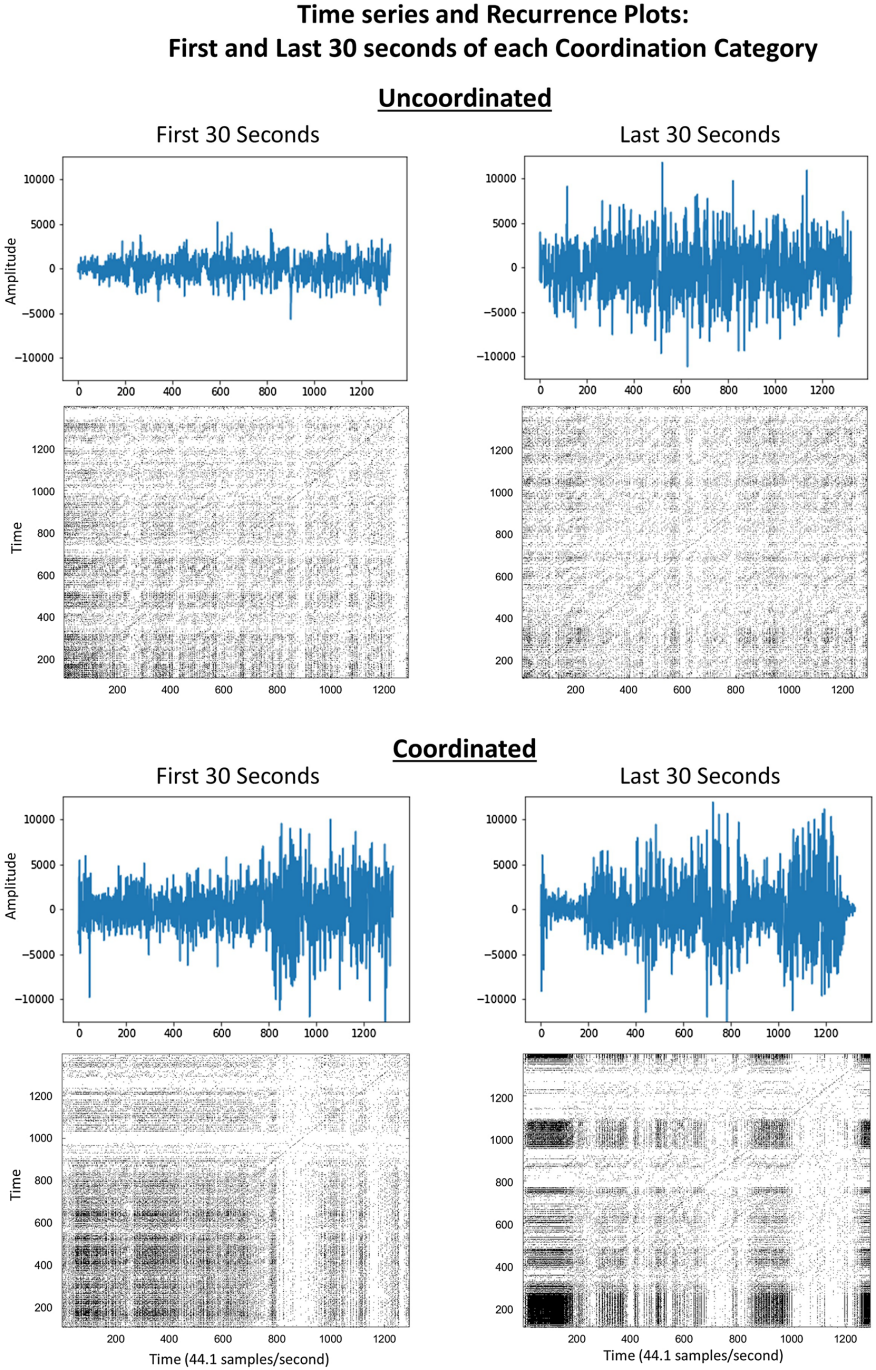
Time series and recurrence plots for the first and last 30 s of each Coordination Category (Uncoordinated or Coordinated). Darker segments of the recurrence plots indicate the presence of more recurrent data points. Vertical and horizontal lines indicate periods of stability in the system, where one state was visited for a period of up to a few seconds at a time. Note: 9 s of audience applause during the Introduction Performance Event were not analyzed, and are subsequently excluded from all data visualizations.

**Figure 2:**
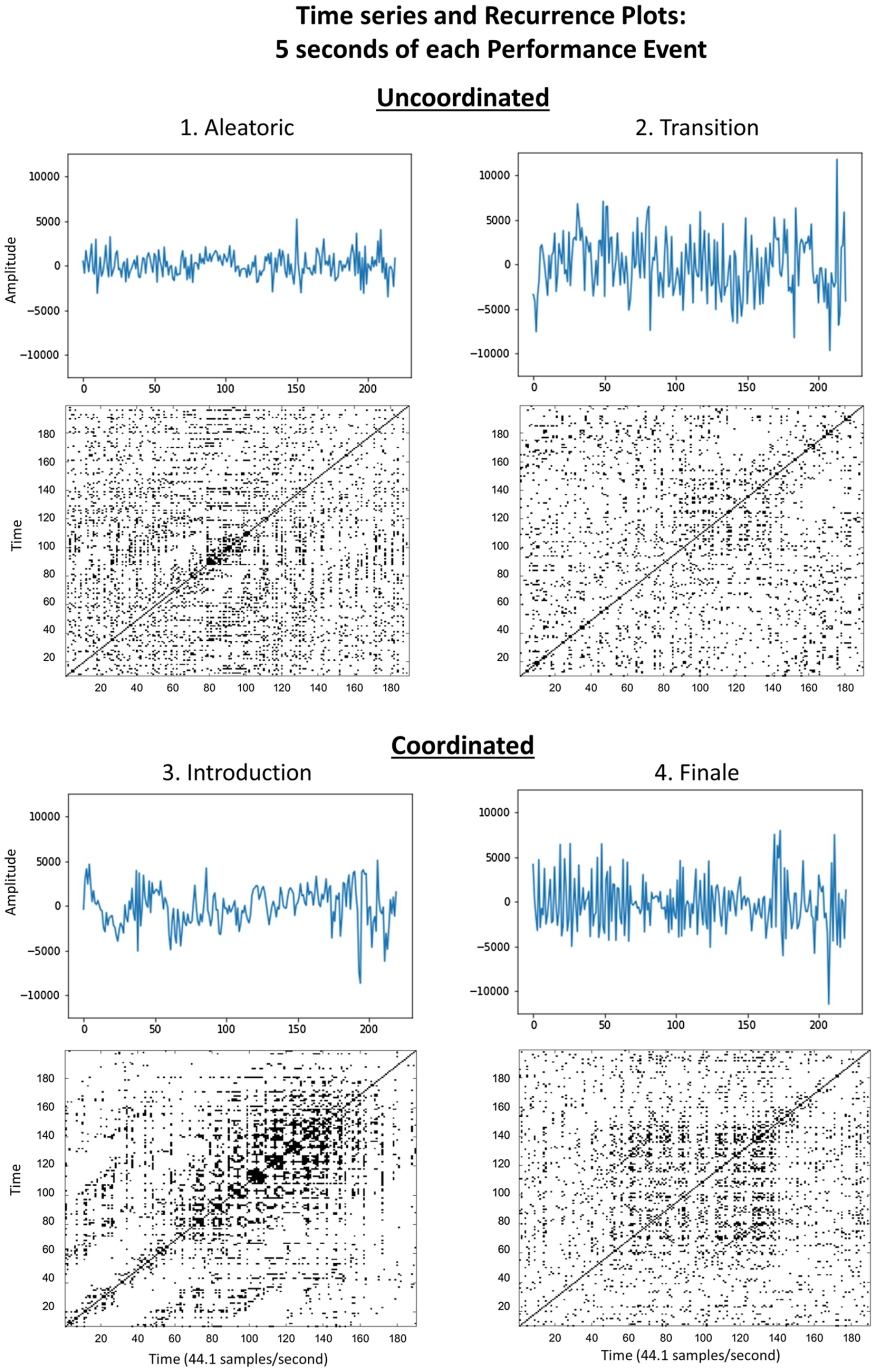
Time series and recurrence Plots for representative 5-s samples drawn from each 30-s sample in Fig. 1, and labeled as the associated Performance Event the 5-s sample is drawn from. Uncoordinated Aleatoric and Transition plots more closely resemble white noise, with fewer recurrent points, shorter diagonals, and less apparent vertical structures. Increased presence of diagonal lines and vertical structures in Coordinated Introduction and Finale plots indicate increasingly coordinated interaction among the musicians. Note: 9 s of audience applause during the Introduction Performance Event were not analyzed, and are subsequently excluded from all data visualizations.

### Recurrence quantification analysis

Figures 1 and 2 show recurrence plots generated from the time series data of the recording. The recurrence plots visualize the characteristic patterns of recurrence which are then quantified through recurrence quantification analysis. Figure 1 displays recurrence plots for the first and last 30 s of each Coordination Category (Uncoordinated or Coordinated). Darker segments of the recurrence plots indicate the presence of more recurrent data points. Vertical and horizontal lines indicate periods of stability in the system, where one state was visited for a period of up to a few seconds at a time.

The recurrence plots in Fig. 2 are representative 5-s samples drawn from each 30 s sample in Fig. 1. These shorter samples are labeled as the associated Performance Event the 5-s sample is drawn from. Note that the “Introduction” section falls in the middle of the performance, marking the shift from the Uncoordinated to Coordinated interaction among the musicians, and thus the order of Performance Events is: 1. Aleatoric, 2. Transition, 3. Introduction, 4. Finale. Thirty second samples from the start and end of each Coordination Category were chosen to maintain balanced samples from each category for computing inferential statistics, and because independent raters noted a clear Transition section in the final 30 s of the uncoordinated performance.

Increased presence of diagonal lines and vertical structures in the Introduction and Finale sections indicate increasingly coordinated interaction among the musicians. The diagonal striping in the Introduction indicates some periodicity in the signal, similar to the periodicity of a sine wave, and results in this case from a(n almost) unison chord during those few seconds. The Aleatoric and Transition plots at the top more closely resemble white noise, with fewer recurrent points, shorter diagonals, and less apparent vertical structures.

**Figure 3:**
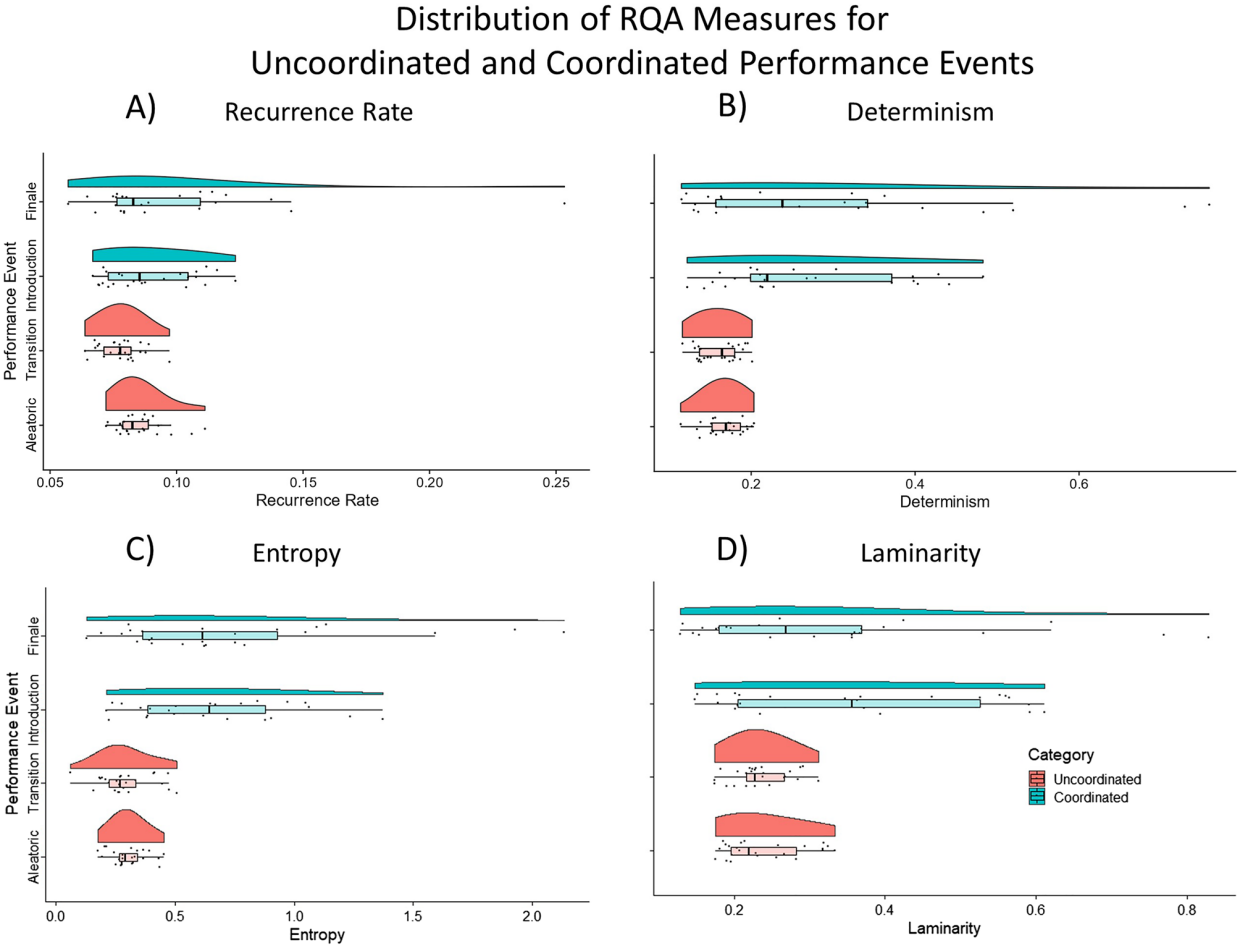
Raincloud plots show higher levels, and wider variance, in each RQA metric in Coordinated compared to Uncoordinated Categories. Boxplots show sample median and interquartile range. Note: 9 s of audience applause during the Introduction Performance Event were not analyzed, and are subsequently excluded from all data visualizations.

**Figure 4:**
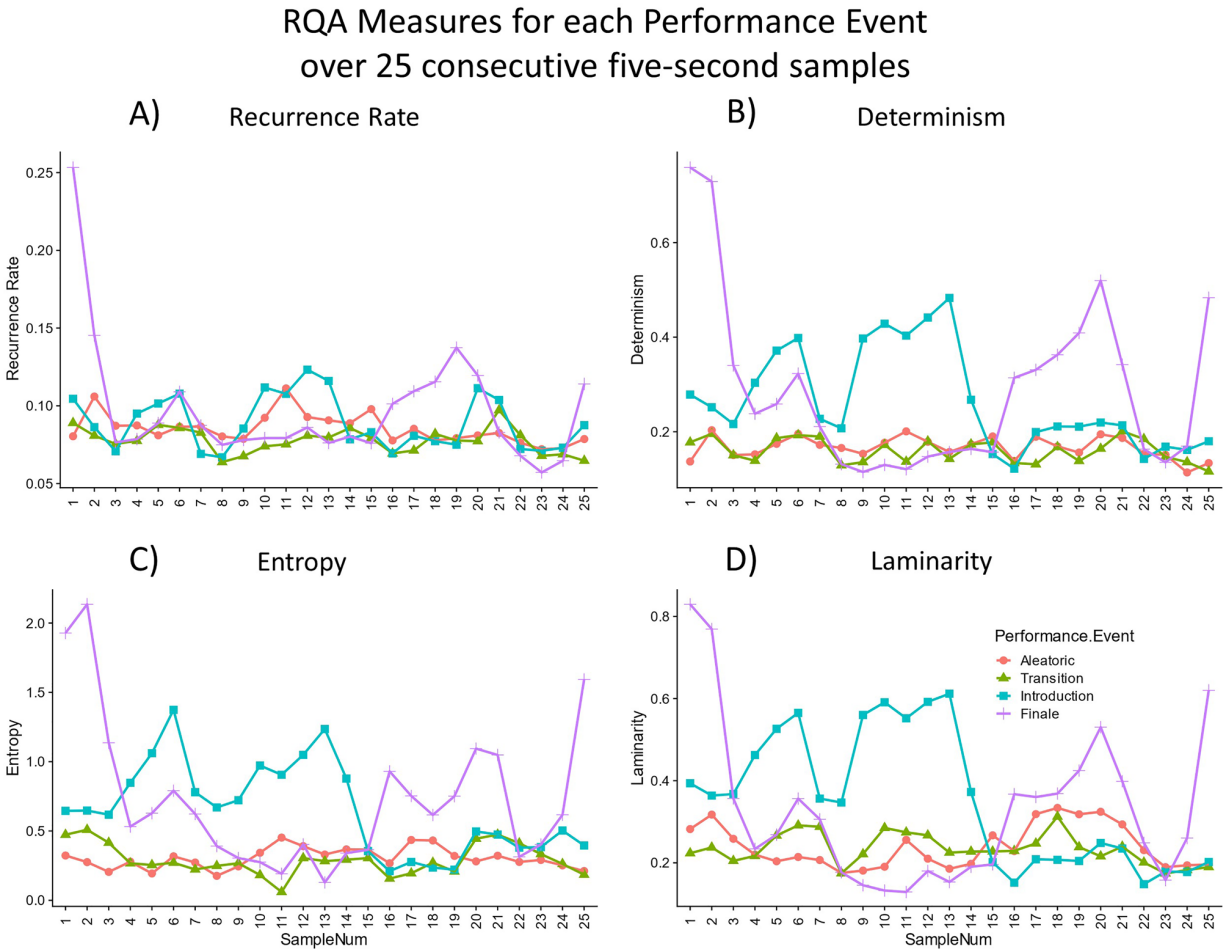
Serial plots visualizing the trajectories of recurrence behaviors over time. (**A**) Coordinated Performance Events (Introduction and Finale) show increased recurrent points overall (Recurrence Rate) compared with Uncoordinated Performance Events (Aleatoric and Transition). (**B**) Emerging presence of longer sequences of behavior as represented by higher levels of Determinism, and (**C**) higher levels of Entropy in Coordinated Performance Events, indicating more variability in sequence length. (**D**) Increased values of Laminarity in Coordinated Performance Events indicate enhanced stability in the system. The intermittency of these stable periods in the Coordinated Performance Events as shown in the recurrence plots (Figs. 1 and 2) can also be seen in the varying high and low values of Laminarity over time in the serial plots. Note: 9 s of audience applause during the Introduction Performance Event were not analyzed, and are subsequently excluded from all data visualizations.

Recurrence Quantification Analysis quantifies the qualitative patterns observed in these recurrence plots. To describe the behavior of our model multi-agent system, five RQA measures were evaluated. The first RQA measures evaluated are common measures of recurrence: (a) patterns that repeat over time (Recurrence, the percentage of recurrent points on the recurrence plot); (b) behaviors that belong to a longer sequence of behavior (Determinism, or the percentage of points that fall on any diagonal line in the recurrence plot); (c) the amount of disorder there is in these sequences (Entropy, or the variability in lengths of these diagonal lines). Additional patterns of stability in the system’s behavior were measured by examining (d) clusters of behavior (Laminarity, or the percentage of points that fall on a vertical line in the recurrence plot), and (e) the average length of time our multi-agent system stays in one behavioral pattern (average diagonal length, the average length of diagonal lines). Average diagonal line length is a measure related to determinism. Longer average diagonal lines reflect the stability of a system by indicating longer, more continuous states. Similarly, higher laminarity shows the rigidity, or “stickiness” of a system that stays in one or more states of a behavior for a length of time^32^.

Higher values and increased variability were evident for most RQA measures for Performance Events within the Coordinated sections of the performance vs Uncoordinated sections (Fig. 3). However, distribution plots do not readily visualize different trajectories of behavior over time. Varying trajectories of each RQA measure for Coordinated vs Uncoordinated sections of the performance is evident in the serial plots in Fig. 4. The majority of RQA values hover around a single value over time during the Uncoordinated Performance Events (Aleatoric and Transition), indicating little interaction among musicians—the agents in our model system. As interactive behavior emerges among musicians, the joint activity of the interacting ensemble in the Coordinated Performance Events (Introduction and Finale) begin to show increased recurrent points overall (Recurrence Rate), with emerging presence of longer sequences of behavior as represented by higher levels of Determinism, and higher levels of Entropy indicating more variability in sequence length. Increased values of Laminarity indicate enhanced stability in the system. The intermittency of these stable periods in the Coordinated Performance Events as shown in the recurrence plots can also be seen in the varying high and low values of Laminarity over time.

### Statistical analysis

#### Descriptive statistics

Statistical analysis was performed on the first and last 25 samples of each Coordination Category, representing the first and last 30 s each of Uncoordinated and Coordinated sections of the piece. Recurrence (REC) for the first 30 s of each category was highest in coordinated (mean 0.093/ sd 0.030) when compared to uncoordinated (mean 0.081/ sd 0.009). REC for Performance Event showed a slight decrease from Aleatoric (mean 0.085 /sd 0.009) to Transition (mean 0.078 / sd 0.008) and increased in both Introduction (mean 0.089/ sd 0.017) and Finale (mean 0.098 /sd 0.039) (Fig. 3A). Determinism (DET) for the first 30 s of each category was, highest in the coordinated condition (mean 0.277/ sd 0.147) when compared to uncoordinated (mean 0.163/ sd 0.024). DET for Performance event shows the same pattern, with a slight decrease from Aleatoric (mean 0.167 /sd 0.023) to Transition (mean 0.160 / sd 0.025), and increases in both Introduction (mean 0.266/ sd 0.106) and Finale (mean 0.288 /sd 0.179) (Fig. 3B). Entropy for the first 30 s of each category was highest in the coordinated condition (mean 0.693/ sd 0.432) when compared to uncoordinated (mean 0.295/ sd 0.091). Entropy for Performance event shows the same pattern, with a slight decrease from Aleatoric (mean 0.305 /sd 0.074) to Transition (mean 0.292 / sd 0.110), and increases in both Introduction (mean 0.654/ sd 0.325) and Finale (mean 0.731 /sd 0.521) (Fig. 3C) Laminarity for the first 30 s of each category was highest in the coordinated condition (mean 0.340/ sd 0.175) when compared to uncoordinated (mean 0.236/ sd 0.045). Laminarity for Performance event shows the same pattern, with a slight decrease from Aleatoric (mean 0.240/ sd 0.053) to Transition (mean 0.234 / sd 0.037), and increases in both Introduction (mean 0.353/ sd 0.162) and Finale (mean 0.326 /sd 0.190) (Fig. 3D).

Higher values and increased variability were evident for most RQA measures for Performance Events within the Coordinated sections of the performance vs Uncoordinated sections (Fig. 3). However, distribution plots do not readily visualize different trajectories of behavior over time. Varying trajectories of each RQA measure for Coordinated vs Uncoordinated sections of the performance is evident in the serial plots in (Fig. 4). The majority of RQA values hover around a single value over time during the Uncoordinated Performance Events (Aleatoric and Transition), indicating little interaction among musicians-the agents in our model system. As interactive behavior emerges among musicians, the joint activity of the interacting ensemble in the Coordinated Performance Events (Introduction and Finale) begin to show increased recurrent points overall (Recurrence Rate), with emerging presence of longer sequences of behavior as represented by higher levels of Determinism, and higher levels of Entropy indicating more variability in sequence length. Increased values of Laminarity indicate enhanced stability in the system. The intermittency of these stable periods in the Coordinated Performance Events as shown in the recurrence plots can also be seen in the varying high and low values of Laminarity over time.

#### Inferential statistics: model comparisons

To further examine the trends above, Linear Mixed Effects models (LMEs) were applied to determine the differential effect of Coordination Category and Performance Event on each RQA measure of interest. LMEs (or multilevel models) account for the nested structure of hierarchical data, as when individual observations are nested within groups^33^. In this case, the individual observations of RQA measures in each 5 s sample are nested within larger uncoordinated or coordinated categories (or the subcategories of performance event). A linear mixed effects model assumes that fixed effects (of coordination category or performance event) do not vary, while the random effect structure of a LME allows each individual sample to vary. This accounts for interdependence between subsequent samples in each category. Model comparisons between LME with and without fixed effects enables inference regarding the contribution of the fixed effect of interest^34^. If model comparisons show that a model with fixed effects is statistically different from a model without a fixed effects (a random effects only model in this case), then we can conclude that the model with the fixed effect better explains the data. Therefore, we can infer the differential effect of the fixed effect of interest (Coordination Category or Performance Event) on the RQA measure of interest.

Log REC was predicted by Coordination Category ($\chi ^2$ (1) = 8.0805, p = 0 .0045), and by Performance Event ($\chi ^2$ (3) = 12.594, p = 0.0056 ). Log DET was predicted by Coordination Category ($\chi ^2$(1) = 30.455, p = 3.418e-8), and by Performance Event ($\chi ^2$ (3) = 30.656, p = 1.004e-6). Log Entropy was predicted by Coordination Category ($\chi ^2$ (1) = 41.261, p = 1.332e-10), and by Performance Event ($\chi ^2$ (3) = 41.739, p = 4.557e-9). Log Laminarity was predicted by Coordination Category ($\chi ^2$ (1) = 10.382, p = .0013), and by Performance Event ($\chi ^2$ (3) = 11.516, p = 0.0092).

LME model comparison results are reported in Table 1 for log transformations of each RQA measure, except for Average Diagonal Length because assumptions of normality/heterocedasticity were not met. LMEs with a fixed effect of Coordination Category showed lower AIC and BIC values than the null model, or the model with a fixed effect of Performance Event, indicating that RQA measures are better predicted by the Coordination Category (Uncoordinated vs Coordinated) of each sample than by the Performance Event (Aleatoric, Transition, Introduction, and Finale—which are smaller subdivisions of each Coordination Category). Table 1. Linear mixed effects model results. Models were fixed effect of interest (Coordination Category or Performance Event) with random effect of Sample Number, against a random intercept model without the fixed effect in question. Models reveal differential effect of the fixed effect of interest on the log transformed RQA measure of interest (Recurrence Rate, Determinism, Entropy, or Laminarity).

**Table 1:** Linear mixed effects models evaluating the effect of Coordination category and performance event on each RQA measure of interest.

Fixed effects	Recurrence rate			Determinism		
	Full model		Null model	Full model		Null model
	Estimate	SE		Estimate	SE	
**Coordination category**						
Uncoordinated (intercept)	− 2.514	0.0304		− 1.8225	0.0503	
Coordinated	0.1052	0.0360		0.4217	0.0703	
**Goodness of fit**						
Deviance	− 43.82		− 35.74	75.801		106.256
AIC	− 35.82		− 29.74	83.801		112.256
BIC	− 25.4		− 21.924	94.222		120.071
$\chi ^2$ (df)	8.0805(1)**			30.455(1)***		
**Performance event**						
Aleatoric (intercept)	− 2.4678	0.0389		− 1.8006	0.0706	
Transition	− 0.0924	0.049		− 0.0439	0.0993	
Introduction	0.0324	0.0494		0.4039	0.0993	
Finale	0.0856	0.0494		0.3956	0.0993	
**Goodness of fit**						
Deviance	− 48.334		− 35.74	75.599		106.256
AIC	− 36.334		− 29.74	87.599		112.256
BIC	− 20.703		− 21.924	103.23		120.07
$\chi ^2$ (df)	12.594(3)**			30.656(3)***		

Fixed effects	Entropy			Laminarity		
	Full model		Null model	Full model		Null model
	Estimate	SE		Estimate	SE	
**Coordination category**						
Uncoordinated (intercept)	− 1.2647	0.0709		− 1.4567	0.0535	
Coordinated	0.7169	0.1003		0.2506	0.0758	
**Goodness of fit**						
Deviance	145.78		187.04	89.655		100.037
AIC	153.78		193.04	97.655		106.037
BIC	164.2		200.86	108.08		113.85
$\chi ^2$ (df)	41.261 (1)***			10.382 (1)**		
**Performance event**						
Aleatoric (intercept)	− 1.2167	0.1001		− 1.4504	0.0753	
Transition	− 0.0961	0.1415		− 0.0127	0.1065	
Introduction	0.6593	0.1415		0.3008	0.1065	
Finale	0.6783	0.1415		0.1877	0.1065	
**Goodness of fit**						
Deviance	145.3		187.04	88.521		100.037
AIC	157.3		193.04	100.52		106.04
BIC	172.94		200.86	116.15		113.85
$\chi ^2$ (df)	41.739 (3)***			11.516 (3)**		

***$$p<0.001$$, **$$p<0.01$$, *$$p<0.05$$.

## Discussion

The current study evaluated a musical performance which enacted a real-life simulation of the transition from uncoordinated to orchestrated (non-emergent) coordinated behavior. That is, the musicians in this ensemble simulated the transition from disorder to order in one form of social interaction—a musical performance–by following the orchestration of the musical score, accentuated by the presence of a conductor on stage at just the time the musicians begin to play music *together* as a single interacting ensemble.

We have empirically demonstrated differences in the acoustic coordination patterns of this originally non-interacting collection of independent musicians versus their collective dynamics as an interdependent group of musicians participating in a joint musical interaction. Thus, this study is one example of how conceptualizing and evaluating musical interaction using the tools of coordination dynamics and dynamical systems theory can reveal insights into the self-organizing behavior which underlies multi-agent musical interaction (see Schiavio et al, 2021 for a thorough review^35^).

Unlike previous studies of human social interaction, this study evaluated acoustic data of a musical performance to infer the global behavior of musicians in the performing ensemble. This means we did not have access to the behavior of individual musicians in order to evaluate correlations between individuals. Instead, we relied on recurrence features extracted from global acoustic data and represented in recurrence plots. The uncoordinated acoustic behavior of individual, non-interacting musicians at the start of the performance demonstrated lower measures of recurrence and stability in the group-level acoustic data. When the musicians began interacting with each other as a coordinated musical ensemble, recurrence and stability measures increased overall. The interaction served as a coupling mechanism for this multi-agent human group, just as the physical platform served as a coupling mechanism for the metronome group. In addition, the interacting ensemble also exhibited further variability in these recurrence measures. This indicates that the interacting ensemble of musicians were able to explore a greater variety of acoustic behavior than when they performed as a stage full of individual musicians.

When the function of each musician becomes interdependent with the functions of the other musicians, the group becomes a complex system. The collective cognition (c.f.^36,37^ ) that takes place in order to generate the coordinated music makes the group function a little bit like one large mind^38,39^ (c.f.^40^ ). Thus, when that collective mind has an audio time series extracted from it (that is subjected to state-space reconstruction),it can provide insight into the dynamics by which those individual subcomponents achieve their coordinated behavior.

We present an example of Recurrence Quantification Analysis applied to group level acoustic data from a single performance. However, this musical performance is a model system for other forms of multi-agent human interaction. A priori, we know that the local rules that govern the emergence of interaction in this ensemble arise from a musical score, which stipulates when the musicians must begin performing as an interacting group, as well as a conductor who acts as a leader during the coordinated section of musical performance. This provides for the ecological validity of a natural performance as well as ground truth knowledge of the ensemble’s acoustic performance as they transition from uncoordinated to coordinated behavior. Thus, RQA applied to this model system provides an indication of the possible expected distributions for what recurrence dynamics to expect in truly emergent coordination in multi-agent human interaction in the wild—perhaps in less orchestrated (i.e. more improvised) forms of musical interaction such as leaderless interaction in free jazz improvisation^41^, and even day-to-day social dynamics extending beyond musical interaction, such as walking in groups^42^ or interacting in large crowds.

Extending the current analysis methods to other forms of multi-agent human interaction will also expand current knowledge regarding the affective dynamics of acoustic and motor coordination during social interaction. Listening to music while moving in time with a partner increases perceived connectedness among a dyad^43^. Movement synchrony in dancers increases affiliation with the group^44^ and can increase affective engagement from an audience^45^. In a dot-motion paradigm, velocity-based synchrony (associated with expert interaction) in comparison with interval-based synchrony (associated with novice interaction) from ostensibly improvising performers is rated by observers as more beautiful, and the ‘performers’ are judged to like each other more^46^. The affiliatory effects of synchronous interaction are not always positive, however, and can actually lead to increased compliance with requests to engage in aggressive behavior^47^. Multi-agent groups in the wild, such as crowds at a sporting event or gathering for a protest, may not be engaging in strictly synchronous motor coordination, however their acoustic behavior may exhibit measurable patterns of distributed coordination which influence the affective states of the group and individual. A question remains as to what are the recurrence dynamics of acoustic behavior of multi-agent groups in the wild, and what role does coordinated acoustic behavior play in the affective dynamics of individuals engaging in or observing these social interactions.

## Methods

### Extracting acoustic data

**Table 2:** Coordination category and performance event labels for recurrence quantification analysis.

Music event	Coordination category	Performance event	Start time	End time	30-s sample
Recording starts	Audience noise	–	0m00s	0m14s	–
Scattered entrances	Uncoordinated	Aleatoric	0m14s	2m43s	0m14s to 0m44s
Flute cue	Uncoordinated	Transition	2m43s	3m15s	2m45s to 3m15s
French horn cue	Coordinated	Introduction	3m15s	3m53s	3m15s to 3m53s*
Conductor appearance	Audience noise	–	3m37s	3m45s	*discarded above
Drum cue	coordinated	–	3m53s	9m06	–
Performance continues	Coordinated	Finale	8m36s	9m06s	8m36s to 9m06s
Performance ends	Audience noise	–	9m06s	9m23s	–

30-s samples used for analysis are indicated. Audience noise was discarded before analysis.

An audio recording of “Welcome to the Imagination World” was obtained from the 2009 performance by the Inagauken Wind Orchestra posted on YouTube. An MP3 was downloaded using the YouTube to Mp3 video converter. The audio recording was labeled by two independent raters with terminal music degrees and substantial training in music theory. Raters were familiarized with the program notes for the composition (retrieved from http://www.windrep.org) and were instructed to identify where the musicians transition from “random ad lib” to “unify[ing] majestic introduction” as described in the program note from the composer (operationalized as *uncoordinated* and *coordinated*, respectively), as well as noting any details of the performance they found important. The audio was subsequently labeled into two Coordination Categories: Uncoordinated and Coordinated. The first and last 30-s of each Coordination Category (Uncoordinated or Coordinated) were also labeled into four Performance Event subcategories (Aleatoric, Transition, Introduction, and Finale), two in each Coordination Category, respectively, as shown in Table 2.

The audio recording was converted from stereo to mono in Audacity 2.3.0, converted from an MP3 to a WAV file, removed DC offset, and normalized to − 1.0 dBFS. Python 3.7 in Jupyter Notebooks was used first to create a time series of the full audio data, then to downsample this time series from 44.1 kHz to 44.1 Hz. Downsampling to this rate prioritizes the rhythmic content and aggregate amplitude of the acoustic signal rather than pitch or harmonic properties for the purposes of Recurrence Quantification Analysis. It may be a concern that this is a low sampling rate in relation to human auditory perception, which is sensitive to pitch information in the 20–20,000 Hz range. This is not problematic, however, as this analysis does not seek to explain pitch perception but rather recurrence properties of sound onsets in the acoustic signal itself. This downsampling filters out sound wave properties interpreted as pitch by the human auditory system while preserving frequencies relevant to rhythm perception and identification of event sequences. A 44.1 Hz sample rate is more than sufficient to capture rhythmic events performed within a tempo range of 60–135 bpm (1–2.25 Hz) as in this performance. Finally, a separate time series was created for each 30-s Coordination Category. The time series for each labeled Performance Event Category was then extracted into 5-s overlapping windows, sliding by 1 s at a time, saving only full 5-s samples. Time series containing only audience noise, or dominated by audience noise, were discarded. This included 9 s within the ‘Introduction’ Coordination Category which were discarded due to noise from audience applause overshadowing the signal from the music. These samples are not included in any analysis or data visualization.

Note: 30 s samples were chosen for analysis due to limitations in duration of uncoordinated performance. The Uncoordinated section of music, at 3 minutes in duration, is half the length of the remaining 6 minutes of the Coordinated section. An equal representation from each Coordination Category is required so as not to bias the results of statistical analysis. We know, due to the score, where the coordinated music begins and ends. Further, independent raters indicated a clear Transition section during the last 30 s of Uncoordinated performance. For this reason we choose to analyze both the first and last 30 s of the shorter Uncoordinated section, and the first and last 30 s of the longer Coordinated section. This selection also allows us to compare recurrence dynamics between the the start and end of each Coordination Category in the case that RQA shows individuals are more coordinated at the end of a section than the beginning after interacting for a period of time, rather than solely because of the coordination indicated by the musical score. For an overview of the global variability of recurrence metrics across a larger subset of data, see Supplementary Fig. S1 for a serial plot visualizing all six 30-s samples from Uncoordinated performance and the first and last three 30-s of Coordinated performance.

### Recurrence quantification analysis

The CRP toolbox in MATLAB 2018b was used to visualize the acoustic time series as recurrence plots and to carry out RQA^48,49^. RQA parameters were set with an embedding dimension of 4, delay of 10, neighborhood size (radius) of 1 $*$ standard deviation, using maximum norm to calculate neighbors of the phase space trajectory. Parameters for the time delay and embedding dimension were chosen based on AMI and FNN respectively using a custom MATLAB GUI provided from the 2019 APA Advanced Training Institute in Nonlinear Methods for Psychological Science. There are various approaches to setting the threshold value for detecting nearest neighbors. In classification based on recurrence dynamics of harmonic, transient, and noisy acoustic signals, Zhang 2011^50^ set this threshold value using 1 $*$ standard error. Here we set the threshold value at 1 $*$ standard deviation, because the standard deviation is always larger than standard error, assuring a radius large enough to sufficiently capture recurrent structures in the recurrence plots. 5 $*$ $\sigma$ has been suggested as an optimal threshold value for detecting signal in cases of high observational noise^51^, however, 1 $*$ $\sigma$ is standard and is preferable when the amount of observational noise is unknown^52^. For further discussion regarding parameter selection in RQA see Marwan, 2011^53^ and Webber and Marwan, 2015^54^.

### Statistical analysis

Statistical analysis was performed on the first and last 25 samples of each Coordination Category, representing the first and last 30 s each of Uncoordinated and Coordinated sections of the piece. Raincloud plots^55^ and serial time series plots to visualize distributions and trajectories of the RQA measures were created in RStudio 1.1.463 using ggplot2^56^.

Linear Mixed Effects models (LMEs) were applied to determine the differential effect of Coordination Category and Performance Event on each RQA measure of interest. LMEs were calculated using the lme4 package^57^. The first model examined the effects of Coordination Category on each RQA measure, with a fixed effect of Coordination Category and random effects of Sample Number, to account for any variance arising from individual 5-s samples. The second model examined the effects of Performance Event on each RQA measure, with a fixed effect of Performance Event and random effects of Sample Number.

- Full model with fixed effect of Category (Coordinated vs Uncoordinated) and random effects of order (sample number):
  $$\begin{aligned} \begin{aligned} {\text {RQA Metric}}_{i}&\sim N \left( \alpha _{j[i]} + \beta _{1}({\text {Category}}), \sigma ^2 \right) \\ \alpha _{j}&\sim N \left( \mu _{\alpha _{j}}, \sigma ^2_{\alpha _{j}} \right) \text {, for SampleNum j = 1,} \dots \text {,J} \end{aligned} \end{aligned}$$
- Null, intercept-only model without the fixed effect of Category:
  $$\begin{aligned} \begin{aligned} {\text {RQA Metric}}_{i}&\sim N \left( \alpha _{j[i]}, \sigma ^2 \right) \\ \alpha _{j}&\sim N \left( \mu _{\alpha _{j}}, \sigma ^2_{\alpha _{j}} \right) \text {, for SampleNum j = 1,} \dots \text {,J} \end{aligned} \end{aligned}$$
- Full model with fixed effect of Performance Event (Aleatoric, Transition, Introduction, Finale) and random effects of order:
  $$\begin{aligned} \begin{aligned} {\text {RQA Metric}}_{i}&\sim N \left( \alpha _{j[i]} + \beta _{1}({\text {Performance.Event}}), \sigma ^2 \right) \\ \alpha _{j}&\sim N \left( \mu _{\alpha _{j}}, \sigma ^2_{\alpha _{j}} \right) \text {, for SampleNum j = 1,} \dots \text {,J} \end{aligned} \end{aligned}$$
- Null, intercept-only model without the fixed effect of Performance Event:
  $$\begin{aligned} \begin{aligned} {\text {RQA Metric}}_{i}&\sim N \left( \alpha _{j[i]}, \sigma ^2 \right) \\ \alpha _{j}&\sim N \left( \mu _{\alpha _{j}}, \sigma ^2_{\alpha _{j}} \right) \text {, for SampleNum j = 1,} \dots \text {,J} \end{aligned} \end{aligned}$$

LMEs with random intercepts such as this are robust to variability in individual subjects, or 5-s samples in this case. This is because random intercept models assume a different baseline-level of the RQA measure of interest in each fixed effect for each sample, thus accounting for any differences that may appear by virtue of the sequential order of obtaining each sample. Mixed models also address issues of non-independence due to inherent correlations between successive samples of musical performance data^33^. Goodness of fit was evaluated by model comparison of the full models against null, intercept only models without the fixed effect in question, as shown in the equations above. Four of the five RQA measures of interest were modeled (Recurrence Rate, Determinism, Entropy, and Laminarity). LME results based on Average Diagonal Length are not reported. Residuals plots revealed that the LMEs for Average Diagonal Length did not meet criteria for assumptions of normality and heteroscedasticity, even after log transformation, and as such were not a good model for the data. Statistical significance was obtained by computing a likelihood ratio test of the full model to a null model without the fixed effect in question.

## Supplementary Information

Supplementary Information.
