# Supplementary material for: Coordination dynamics of multi-agent interaction in a musical ensemble
Source: Sci Rep. 2022 Jan 10;12:421. doi: 10.1038/s41598-021-04463-6 (PMC8748883; doi:10.1038/s41598-021-04463-6)
Supplement: Supplementary file 1 — Supplementary Information. [file 41598_2021_4463_MOESM1_ESM.pdf]

Supplemental Materials: Coordination Dynamics of Multi-Agent Interaction in a Musical Ensemble

Shannon Proksch[1,*], Majerle Reeves[2], Michael Spivey[1], and Ramesh Balasubramaniam[1]

[1]University of California-Merced, Cognitive and Information Sciences, Merced, USA
[2]University of California-Merced, Applied Mathematics, Merced, USA
[*]sproksch@ucmerced.edu

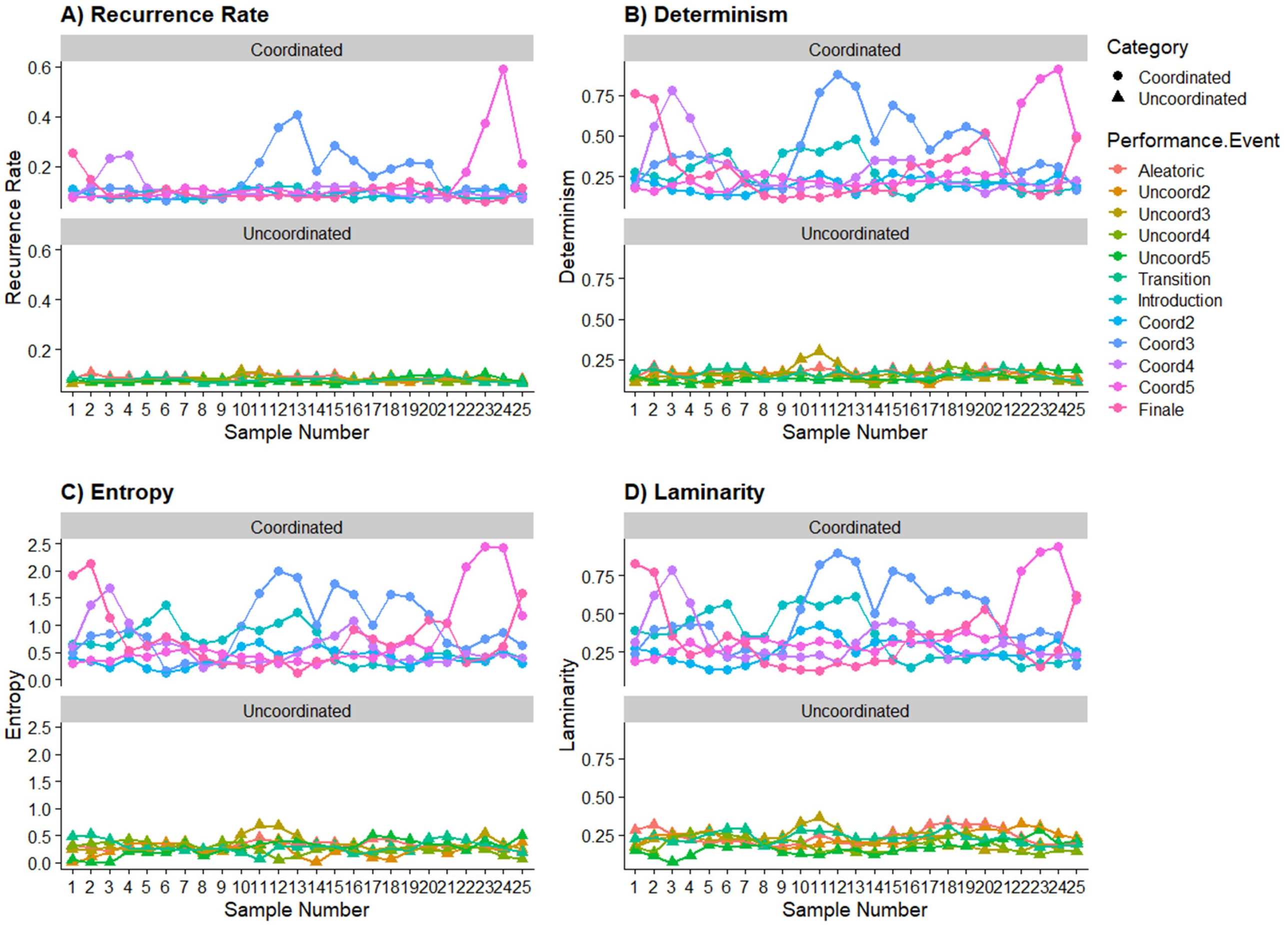

Supplementary Figure 1. Serial plots visualizing the trajectories of recurrence behaviors over time. Shown are all six 30-samples of Uncoordinated performance, and the first and last three 30-second samples of Coordinated performance. Recurrence and stability measures are increased overall and exhibit greater variability during Coordinated compared to Uncoordinated performance.
